# Effect of low-level laser therapy on the expression of inflammatory mediators and on neutrophils and macrophages in acute joint inflammation

**DOI:** 10.1186/ar4296

**Published:** 2013-09-12

**Authors:** Ana Carolina Araruna Alves, Rodolfo de Paula Vieira, Ernesto Cesar Pinto Leal-Junior, Solange Almeida dos Santos, Ana Paula Ligeiro, Regiane Albertini, Jose Antonio Silva Junior, Paulo de Tarso Camillo de Carvalho

**Affiliations:** 1 Postgraduate Program in Rehabilitation Sciences, Universidade Nove de Julho (UNINOVE), Rua Vergueiro 235, 01504-001 São Paulo, SP, Brazil; 2 Postgraduate Program in Biophotonics Applied to Health Sciences, Universidade Nove de Julho (UNINOVE), Rua Vergueiro 235, 01504-001 São Paulo, SP, Brazil; 3 Department of Physical Therapy, Universidade Nove de Julho (UNINOVE), Rua Vergueiro 235, 01504-001 São Paulo, SP, Brazil

## Abstract

**Introduction:**

Inflammation of the synovial membrane plays an important role in the pathophysiology of osteoarthritis (OA). The synovial tissue of patients with initial OA is characterized by infiltration of mononuclear cells and production of proinflammatory cytokines and other mediators of joint injury. The objective was to evaluate the effect of low-level laser therapy (LLLT) operating at 50 mW and 100 mW on joint inflammation in rats induced by papain, through histopathological analysis, differential counts of inflammatory cells (macrophages and neutrophils), as well as gene expression of interleukin 1-beta and 6 (IL-1β and IL-6), and protein expression of tumor necrosis factor alpha (TNFα).

**Methods:**

Male Wistar rats (*n *= 60) were randomly divided into four groups of 15 animals, namely: a negative control group; an inflammation injury positive control group; a 50 mW LLLT group, subjected to injury and treated with 50 mW LLLT; and a 100 mW LLLT group, subjected to injury and treated with 100 mW LLLT. The animals were subject to joint inflammation (papain solution, 4%) and then treated with LLLT (808 nm, 4 J, 142.4 J/cm^2^, spot size 0.028 for both groups). On the day of euthanasia, articular lavage was collected and immediately centrifuged; the supernatant was saved for analysis of expression of TNFα protein by enzyme-linked immunosorbent assay and expression of IL-1β and IL-6 mRNA by real-time polymerase chain reaction. A histologic examination of joint tissue was also performed. For the statistical analysis, analysis of variance with Tukey's *post-hoc *test was used for comparisons between each group. All data are expressed as mean values and standard deviation, with *P *< 0.05.

**Results:**

Laser treatment with 50 mW was more efficient than 100 mW in reducing cellular inflammation, and decreased the expression of IL-1β and IL-6. However, the 100 mW treatment led to a higher reduction of TNFα compared with the 50 mW treatment.

**Conclusions:**

LLLT with 50 mW was more efficient in modulating inflammatory mediators (IL-1β, IL-6) and inflammatory cells (macrophages and neutrophils), which correlated with the histology that showed a reduction in the inflammatory process.

## Introduction

Osteoarthritis (OA) is a disease that involves damage to the cartilage and the subchondral bone. The concept that synovial inflammation contributes to the development of OA is relatively recent (1990), and since then has been gaining strength [1].

Histological changes seen in the synovial membrane in OA generally include characteristics indicating an inflammatory synovitis; more specifically, the changes include a variety of abnormalities such as synovial lining hyperplasia, infiltration of macrophages and lymphocytes, neoangiogenesis, and fibrosis [2]. Inflammation of the synovial membrane plays a key role in the pathophysiology of OA. Immunohistochemical analysis confirmed that the synovial tissue of patients with early OA is characterized by infiltration of mononuclear cells and production of proinflammatory cytokines and other mediators of articular damage [3].

Several studies indicate that macrophages are involved in OA pathophysiology, where they produce growth factors such as vascular endothelial growth factor and inflammatory cytokines such as interleukin (IL)-1β and tumor necrosis factor alpha (TNFα), and that cytokines produced by macrophages may amplify inflammation in joints [4-7]. This inflammation induces synovial cells to produce additional cytokines and chemokines as well as matrix metalloproteinases. Moreover, macrophages present in the synovial fluid of OA express various receptors that mediate the inflammatory cascade [8].

Synovial inflammation is an important source of both proinflammatory and anti-inflammatory mediators, and these have a role in OA. IL-1β and TNFα produced by synovial cells induce a cascade of degradation, leading to joint injury. These mediators, in particular, interleukins (IL-1β and TNFα), chemokines, growth factors, and matrix metalloproteinases are found in the synovial fluid (synthesized by synoviocytes, chondrocytes, and infiltrating leukocytes) and they affect the cellular function of articular tissues [9-13].

According to Pelletier and colleagues, the main goals for the control of OA are to reduce symptoms, minimize disability, and limit the progression of structural changes [14]. The understanding of the role of catabolic factors in cartilage degradation, as well as the effects of synovial inflammation and cytokines on disease progression, has improved substantially in the past two decades. This knowledge provides the framework necessary to design strategies for the control of this articular disease.

Given the above, the search for effective therapies has focused on treatment modalities that modulate the expression of these mediators [15-17]. Low-level laser treatment (LLLT) therapy has great potential utility in this regard, since several studies have shown that it can regulate interleukin and inflammatory mediator expression [18-23] and can also reduce inflammatory signs and symptoms that are present in osteoarthritis [24-26].

The present study therefore aimed to evaluate the effect of LLLT operated at 50 mW and 100 mW and using identical laser parameters (except for power density and time of irradiation) on acute joint inflammation induced by infiltration of 4% papain in the rat knee.

## Materials and methods

### Animals

The sample population consisted of 60 male Wistar rats (*Norvegicus albinus*), 90 days old, weighing 250 to 300 g. The animals were obtained from the animal housing facility of the Universidade Nove de Julho (Brazil) and were kept under controlled conditions of light and temperature, with free access to water and chow. All experimental procedures were approved by the Institutional Research Ethics Committee (AN 0016/2011), and were according to the guidelines of the Brazilian College for Animal Experimentation as well as the standards of the International Council for Laboratory Animal Science.

### Experimental groups

Sixty animals were randomly distributed into four groups of 15 animals each. The first group (control) did not receive any kind of intervention; the second group (injury), received induction but did not receive any treatment; the third group (LLLT 50 mW) was treated with LLLT at 50 mW; and rats of the fourth group (LLLT 100 mW) were treated with LLLT at 100 mW. All the groups were evaluated 24 hours post injury (five animals per group, at each experimental time point).

### Papain-induced inflammation

The animals were anesthetized with an intramuscular injection of a 7% ketamine solution (Cetamin; Syntec, Cotia, SP, Brazil) and 0.3% xylene solution (Xilazin; Syntec) at a proportion of 2:1 (0.2 ml/100 g). The induction of OA was then performed following previously published methods [27,28]. Specifically, 200 μl injections were administered in the right knee of the hind leg of each animal with a 4% papain solution dissolved in 10 ml saline solution, to which 10 ml cysteine solution (0.03 M) was added. This solution was used as the activator to produce cartilage injury. The animals were then immediately submitted to the administration of LLLT.

### Low-level laser therapy

An arsenide and aluminum gallium-type diode laser with a wavelength of 808 nm from Photon Laser III DMC (Sao Carlos, SP, Brazil) was used. The optical power was calibrated using a Newport multifunction optical meter (Model 1835C; Newport Corporation, Irvine, CA, USA). The dose and parameters are summarized in Table 1.

**Table 1:** Low-level laser therapy parameters

Group	**λ (nm)**	Type of diode laser	Mean power output (mW)	Spot size (cm^2^)	Power density (W cm^2^)	Energy (J)	Energy density (J cm^2^)	Time per point (seconds)
50 mW LLLT	808	GaAlAs	50	0.028	1.78	4	142.4	80
100 mW LLLT	808	GaAlAs	100	0.028	3.57	4	142.4	40

GaAlAs - arsenide and aluminum gallium; λ, - wavelength; LLLT - low-level laser therapy.

### Irradiation

Laser irradiation was given transcutaneously at two points: medial and lateral. Laser irradiation was performed immediately after the papain-cysteine injection, on the right knee in groups at power output of 50 and 100 mW. The control and injury groups received no treatment and served as the negative and positive control groups, respectively, for the comparative histomorphometric analysis. Animals were immobilized by means of grip and were irradiated at an angle of 90° at the surface of the tissue.

### Sample collections

After receiving the treatment, on the day of euthanasia a procedure for obtaining the washed articular synovial fluid was performed. The articular cavity was washed with 1 ml physiologic serum into the intracapsular knee space, the material was immediately centrifuged at 1,500 rpm/5 minutes, as described previously [29], and the supernatant was stored at -80°C for analysis of inflammatory mediators.

### Total and differentiated cell counts

Articular synovium was washed by injecting 200 μl phosphate-buffered saline (PBS) in the joint cavity, because much material (washed) was recovered (usually around 20 to 30 μl). The recovered lavage was centrifuged at 1,500 rpm for 5 minutes, and the cell pellet was suspended in 200 μl PBS. Total cells were then counted in a hemocytometer (Neubauer chamber, 10 μl; diluting solution, Turk's solution 1:5). The remaining articular material was washed thoroughly and used for the differential cell count (neutrophils, eosinophils, macrophages, and lymphocytes). For this counting, the washed joint was processed in a cytocentrifuge (Eppendorf, Hamburg, Germany) at 450 rpm for 6 minutes, and the slides containing the cell pellet were stained with Diff-Quick. As shown in previous studies, 300 cells were counted on each slide [29,30].

### Evaluation of inflammatory mediators (IL-1β, IL-6)

The amount of IL-1β and TNFα in washed articular synovium was quantified using the enzyme-linked immunosorbent assay, as per the manufacturer's instructions (R&D Systems, Minneapolis, MN, USA.). For this purpose, 96-well plates were coated with 100 ml monoclonal antibody for each cytokine: anti-IL-1β and TNFα diluted in sodium carbonate buffer (0.1 M, pH 9.6). The plates were incubated (4°C) for 18 hours. For blocking, the plates were washed four times with PBS containing 0.05% Tween 20 and then filled with 300 μl/well of blocking solution (3% gelatin in PBS containing 0.05% Tween 20 (Sigma, St. Louis, MO, USA) at 37°C for 3 hours before being subjected to a new cycle of washes. Next, 100 ml properly diluted samples or standards of recombinant cytokines were added to the plate and incubated for 18 hours at 4°C. After washing, 100 μl respective biotinylated antibodies specific for the detection of each cytokine was added and left for 1 hour at room temperature. After washing the plates, the volume of 100 μl streptavidin-peroxidase was added and left for 1 hour at room temperature (22°C) followed by further washes. The reaction was visualized by adding 100 μl/well solution of 3,3',5,5'-tetramethylbenzidine and stopped by adding 50 μl/well sulfuric acid (2 N). The reading was performed in a Spectrum Max Plus 384 spectrophotometer (Molecular Devices Corporation, Sunnyvale, CA, USA) at a wavelength of 450 nm with correction at 570 nm. The sample concentrations were calculated from standard curves obtained with recombinant cytokines. The limit of detection for IL-1β and TNFα was 1.95 pg/ml, while that for IL-6 was 3.13 to 300 pg/ml [19].

### Quantitative reverse transcriptase-polymerase chain reaction

One microgram of total RNA was used for cDNA synthesis and real-time polymerase chain reaction (PCR) gene expression analysis. Initially, contaminating DNA was removed using DNase I (Invitrogen Life Technologies, Carlsbad, CA, USA) at a concentration of 1 unit/μg RNA in the presence of 20 mM Tris-HCl, pH 8.4, containing 2 mM MgCl_2 _for 15 minutes at 37°C, followed by incubation at 95°C for 5 minutes for enzyme inactivation. Reverse transcription was then carried out in a 200 μl reaction mixture in the presence of 50 mM Tris-HCl, pH 8.3, 3 mM MgCl_2_, 10 mM dithiothreitol, 0.5 mM dNTPs, and 50 ng random primers with 200 units of Moloney murine leukemia virus-reverse transcriptase (Invitrogen). The reaction conditions were as follows: 20°C for 10 minutes, 42°C for 45 minutes, and 95°C for 5 minutes.

The reaction product was amplified by real-time PCR on the 7000 Sequence Detection kit using the SYBRGreen core reaction system (ABI Prism, Applied Biosystems, Foster City, California, USA). The thermal cycling conditions were as follows: 50°C for 2 minutes, then 95°C for 10 minutes, followed by 40 cycles at 95°C for 15 seconds and 60°C for 1 minute. Experiments were performed in triplicate for each data point.

IL-10 and IL-1β mRNA abundance was quantified as a relative value compared with an internal reference, β-actin, whose abundance did not change between the varying experimental conditions. Primers used for real^-^time PCR are as follows: IL-6 [GenBank:E02522], forward primer 5'-TCCTACCCCAACTTCCAATGCTC-3' and reverse primer 5'-TTGGATGGTCTTGGTCCTTAGCC-3'; and IL-1 [GenBank:M98820], forward primer 5'-CACCTCTCAAGCAGAGCACAG-3' and reverse primer 5'-GGGTTCCATGGTGAAGTCAAC-3' [20].

### Euthanasia

At the end of each treatment, animals of each group were identified, weighed, and subsequently euthanized by inhalation of carbon dioxide. This method confers rapid loss of consciousness in response to hypoxia attributed to depression of vital centers and is performed in a carbon dioxide chamber. The tibio-femoral articulation of the right hind leg of each animal was separated for analysis of the cartilaginous tissue of the knee. The material was immediately fixed using a 10% buffered formaldehyde solution and submitted to histological procedures.

### Histological procedures and histopathological analysis

The material was decalcified with ethylenediamine tetraacetic acid and submitted to the classic histological method for embedment in paraffin, dehydration in increasing concentrations of alcohol, clearing with xylol in order to allow the penetration of paraffin, impregnation in paraffin baths and insertion in molds, cross-sectional cuts to a thickness of 5 μm, and finally mounting on a synthetic balsam. Sections were then stained with hematoxylin and eosin to perform histopathological analysis.

### Statistical analysis

The data were tabulated using Microsoft Excel 2007 software and initially assessed for normality using the Shapiro-Wilk test. As normal distribution was observed, analysis of variance with Tukey's *post-hoc *test was used for comparisons between 7, 14, and 21 days within each group as well as between the control, injury, 50 mW LLLT, and 100 mW LLLT groups. All data are expressed as mean and standard deviation values. The GraphPad Prism 5 software program (GraphPad Software, San Diego, CA, USA) was used, with significant difference from the null hypothesis considered when *P *< 0.05.

## Results

### Differential inflammatory cell counting

**Figure 1:**
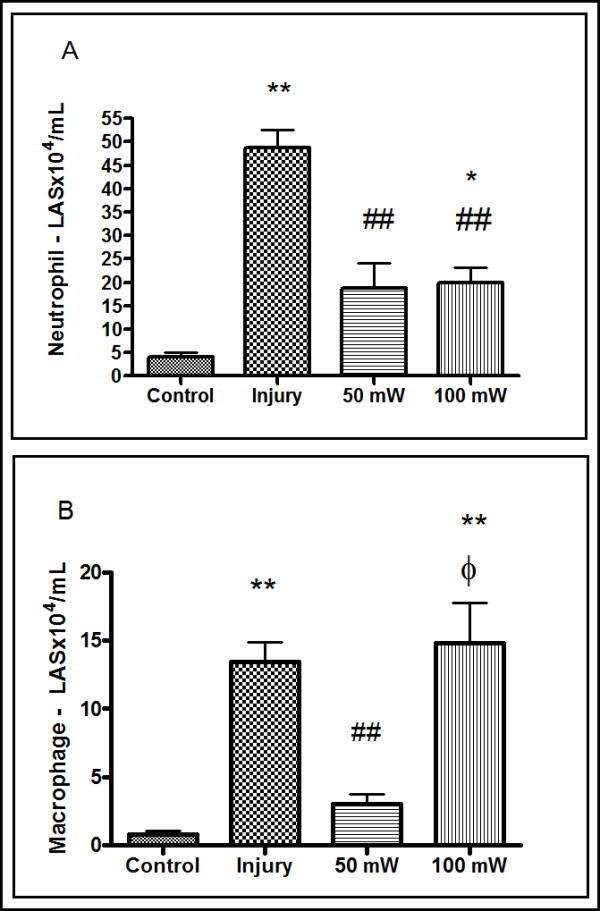
**Analysis of the articular lavage 24 hours after induced papain injury**. **(A) **Absolute neutrophil count calculated from a differential count determined in cytospin preparations stained with the Diff-Quik stain. *P < 0.05 and **P < 0.001, Tukey's test compared with the control group. ##*P *< 0.001, Tukey's test compared with the injury group. **(B) **Absolute macrophage count calculated from a differential count determined in cytospin preparations stained with Diff-Quik stain. **P < 0.001, Tukey's test compared with the control group. ##*P *< 0.001, Tukey's test compared with the injury group. ξ*P *< 0.05 Tukey's test compared with the 50 mW low-level laser therapy group. Results expressed as mean ± standard error of the mean. LAS: Washed Articular Synovium.

The data presented show the total count of viable cells in washed joint fluid that were enumerated in the Neubauer chamber. The control group had fewer cells (6.8 ± 0.5) than the injury group (72.8 ± 5.3) whereas the 50 mW LLLT group (32.3 ± 0.8) and the 100 mW LLLT group (45.8 ± 2.3) had intermediate values. In the differential analysis for neutrophils, the groups treated with 50 mW LLLT (18.88 ± 5.3) and 100 mW LLLT (19.88 ± 3.2) showed a reduction in the absolute number of neutrophils compared with the injury group (48.7 ± 7.3); however, only the 50 mW group showed no statistical difference (*P *> 0.05) when compared with the control group (4.1 ± 0.9). This clearly demonstrates that LLLT reduced the number of neutrophils to approximately the level observed in the control group (Figure 1A).

In Figure 1B the effect of LLLT on the differential count of macrophages can be seen. By statistical analysis (analysis of variance) we found statistical difference for the injury group and 100 mW LLLT group compared to control group (*P *< 0.05)., The 50 mW LLLT group has significant difference compared to injury group and 100 mW LLLT group (*P *< 0.05). No difference was observed between 50 mW LLLT group and control group (*P *> 0.05).

### Effect of LLLT on IL-1β mRNA expression in articular synovial lavage

**Figure 2:**
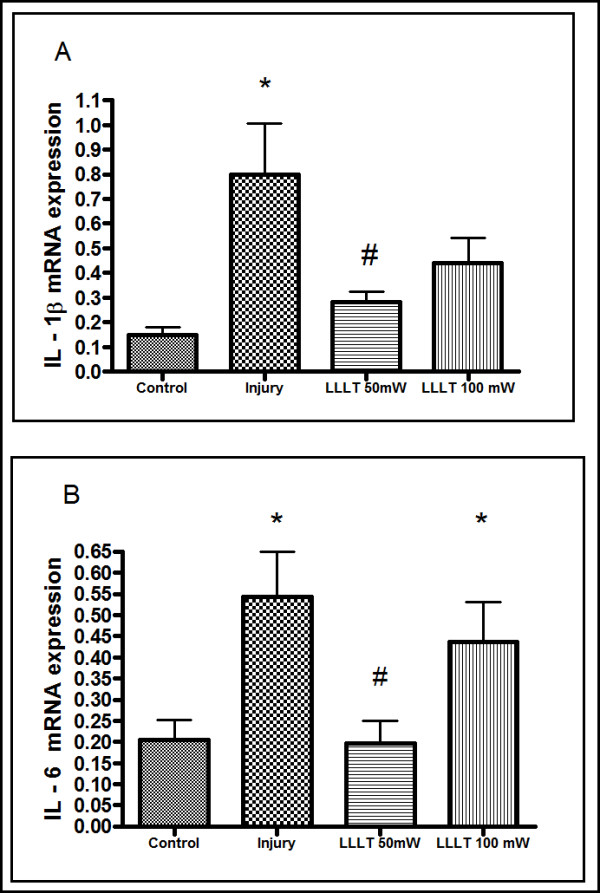
**Effect of low-level laser therapy on IL-1β and IL-6 RNA expression in articular lavage fluid**. **(A) **IL-1β expression measured by real-time polymerase chain reaction (PCR) in articular wash fluid. *P < 0.05, Tukey's test compared with the control group. *#P *< 0.05, Tukey's test compared with the injury group. **(B) **IL-6 RNA expression measured by real-time PCR of articular lavage fluid. *P < 0.05, Tukey's test compared with the control group. #*P *< 0.05, Tukey's test compared with the injury group. Results expressed as mean ± standard error of the mean. LLLT, low-level laser therapy.

In Figure 2A, we can observe that there is a significant difference between the control group and the group challenged with joint injury (*P *< 0.05) in expression of IL-1β. Treatment with 50 mW LLLT reduced the expression of IL-1β to values similar to the control group, and significantly lower than the injury group (*P *< 0.05). In the 100 mW LLLT group, despite not having shown a statistically significant difference (*P *> 0.05) compared with the control group, there was a reduction in IL-1β expression in this treatment group.

### Effect of LLLT on IL-6 mRNA in articular synovial lavage

Figure 2B represents the effect of LLLT on IL-6 mRNA expression. In these experiments, IL-6 expression was significantly increased after papain injection when compared with animals from the control groups. Only treatment with 50 mW LLLT was markedly efficient in reducing papain-induced IL-6 mRNA expression.

### Effect of LLLT on the protein expression of TNFα in articular synovial lavage

**Figure 3:**
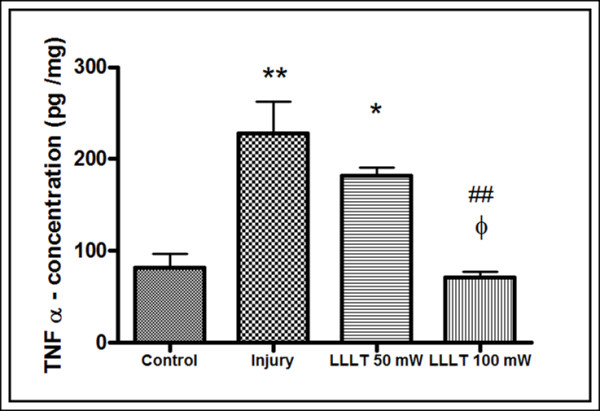
**TNFα concentration in articular lavage fluid 24 hours after induction of papain injuries**. Comparison of mean and standard deviation of the concentration of tumor necrosis factor alpha (TNFα) obtained by enzyme-linked immunosorbent assay in articular lavage fluid 24 hours after induction of papain injuries. *P < 0.05 and **P < 0.001, Tukey's test compared with the control group. ##*P *< 0.001, Tukey's test compared with the injury group. ξ*P *< 0.05, Tukey's test compared with the 50 mW low-level laser therapy (LLLT) group.

Figure 3 shows the results of analysis of protein expression of TNFα in the articular synovial lavage. We can observe that the injury inflammation induced by papain led to a statistically significant increased expression of TNFα compared with the control group and the 50 mW LLLT group (*P *< 0.05). However, the 100 mW LLLT group showed a significantly lower expression of TNFα compared with the injury group (*P *< 0.001), similar to values in the control group.

### Histological analysis

**Figure 4:**
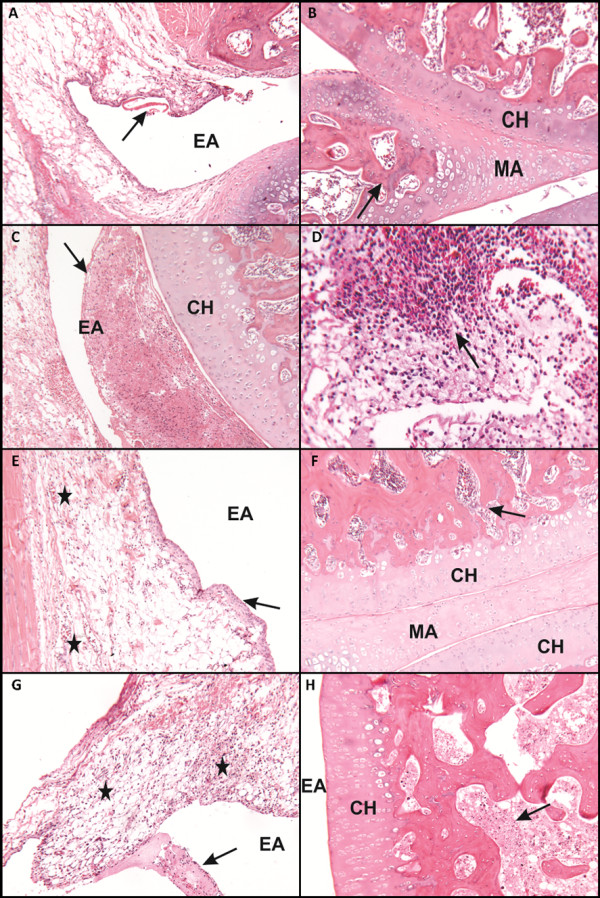
**Photomicrographs of histological preparations 24 hours after induction of the inflammation process with 4% papain**. **(A) **Slight presence of hyaline material in the joint space due to plasma extravasation (arrow). **(B) **Joint surfaces under conditions of normal meniscus and articular signs of ossification (arrow). **(C) **Intense acute inflammatory exudate filling the part of the joint space (arrow). **(D) **Neutrophil accumulation in the connective tissue underlying the synovial membrane (arrow). **(E), (F) **Specimens of normal tissue: (E) intimal layer of the synovial membrane presents usual thickness (arrow), whereas few inflammatory cells are observed in the isolated underlying layer (star); (F) meniscus and articular joint surfaces show signs of tissue integrity. **(G) **Presence of both the acute inflammatory exudate synovial membrane (star) as well as in the joint space and an association with fibrin hyaline material (arrow). **(H) **Cellular degenerative process in the marrow spaces of the epiphyses of the joint (arrow). Stained with hematoxylin and eosin 40×. Scale bar, 20 μm. CH: Hyaline Cartilage; EA: Articular Space; MA: Articular Meniscus.

Upon histological examination, the control group had general features that were consistent with synovial joint normality. The joint spaces did not have inflammatory exudate, and the synovial membranes had thickened intima and subintima showing typical characteristics. The surfaces of coated articular hyaline cartilage were homogeneous, and there was active endochondral ossification of the epiphysis. The control group also had epiphyseal bone marrow with a normal pattern that was filled with red bone marrow. The articular meniscus consisted of thick collagen fibers and chondrocytes, and ossification was present (Figure 4A, B).

In the injury group (induction of the inflammatory process without any treatment) the synovial joint showed acute inflammatory process. The joint spaces had fibrinous and hyaline material that adhered to the surface of the synovial membrane, and some areas had an acute inflammatory infiltrate. The synovial membrane had an intima with normal thickness, but the subintimal layer showed signs of acute inflammatory infiltrate and dilated blood vessels. Similar to that in the control group, the surfaces coated with articular hyaline cartilage were homogeneous and the epiphyseal bone marrow had the normal fill of red bone marrow. Moreover, the epiphysis of the bone marrow presents antagonistic in the process of cellular degeneration (Figure 4C, D).

The group treated with 50 mW LLLT showed synovial joints with few morphological changes, such as high amounts of fibroblast cells and presence of discrete inflammatory cells (a few neutrophils) in the underlying connective tissue of the anterior cruciate ligament (that is, the joint capsule; Figure 4E, F).

The group treated with 100 mW LLLT showed signs of an acute inflammatory process in the synovial joint. The articular spaces were filled with acute inflammatory infiltrate (exudate) and the presence of red blood cells and hyaline material. Apart from this, the synovial membrane had an intima of normal thickness. The layer subintima showed signs of acute inflammatory infiltration and dilated blood vessels. Surfaces coated with articular hyaline cartilage were homogeneous, and the epiphyseal bone marrow had a normal pattern and was filled with red bone marrow. The articular meniscus consisted of thick collagen fibers and chondrocytes, as shown in Figure 4G, H.

## Discussion

OA is not a simple wear-and-tear phenomenon, but an active process that is a part of the reparative response to injury. The disease affects not only the cartilage but also the entire joint structure, including the subchondral bone, synovium, and joint capsule. The exact cause of OA is not yet known. Studies have indicated that inflammation of the synovium may play an important role in the pathogenesis of OA. Proinflammatory cytokines, particularly IL-1β and TNFα, are synthesized by synoviocytes, chondrocytes, and infiltrating leukocytes during the disease process [12].

Several studies have demonstrated the effectiveness of LLLT in treating the inflammatory process, and these studies indicate the modulating property of laser light towards anti-inflammatory and proinflammatory mediators [19-21,23]. However, other studies have shown a failure of LLLT in conditions involving joint damage [31,32].

In this study, we used the protocol of Murat and colleagues for OA [28]. However, we performed an evaluation of the inflammatory changes after 24 hours of induction with papain at 4%, and compared the results of LLLT operating at powers of 50 mW and 100 mW. We used histological analysis and differential counts of inflammatory cells, with an emphasis on neutrophils and macrophages, given that macrophages are the key source of inflammatory cytokines [5-8] that act at the beginning of damage to articular cartilage.

According to Kennedy and colleagues, synovial macrophages are one of the resident cell types in the synovial tissue and while they remain relatively quiescent in the healthy joint, they become activated in the inflamed joint and, along with infiltrating monocytes/macrophages, regulate the secretion of proinflammatory cytokines and enzymes involved in driving the inflammatory response and joint destruction [6]. Synovial macrophages are positioned throughout the sub-lining layer and lining layer at the cartilage-pannus junction and they mediate articular destruction.

We also performed an analysis of gene expression of the cytokines IL-1β and IL-6 and TNFα protein expression, since several studies have shown that inhibition of these cytokines may interfere with the degeneration of articular cartilage and subchondral bone in OA and rheumatoid arthritis [14-16].

Our results show a reduction in both IL-1β and IL-6 expression at the two LLLT operating potencies. However, 50 mW LLLT led to a statistically significant reduction in expression of IL-1β and IL-6 to values obtained in the control group. This observation is consistent with several studies that show modulation of these cytokines by LLLT [18,23,33]. Pallotta and colleagues conducted a study to examine the response to 100 mW LLLT at different times and doses in a model of joint inflammation induced by kaolin plus carrageenan. The authors analyzed several inflammatory mediators, including IL-1β and IL-6, and concluded that LLLT acts by modulating the inflammatory process, and possibly stimulates the production of anti-inflammatory mediators [23].

We also observed in our study that the two groups treated with LLLT (50 mW and 100 mW) had a lower neutrophil count compared with the injury group, similar to those obtained in the control group, indicating that LLLT was able to reduce the migration of neutrophils in the initial inflammatory phase. This was also observed in the histological analysis, although these results indicate a higher efficiency of 50 mW LLLT treatment in attenuation of the general inflammatory process.

These results point to an increase in the modulation of cytokines and inflammatory cells (neutrophils and macrophages), when irradiated by LLLT operating at 50 mW. However, we cannot discard the results of the 100 mW group, primarily with regard to the expression of TNFα.

A study by Pezelj-Ribaric and colleagues in subjects with burning mouth syndrome before and after treatment with LLLT (685 nm continuous wave, 30 mW output power, 3.0 J/cm^2^) that analyzed the expression of TNFα and IL-6 in saliva showed a statistically significant reduction in salivary levels of both after treatment with LLLT [33].

Guo and colleagues performed a study that evaluated and compared the effects of millimeter waves, pulsed electromagnetic fields, ultrasound, LLLT, and short-wave diathermy on the serum levels of TNFα, chondrocyte apoptosis, caspase-3 and caspase-8, in an experimental model of OA in rabbit knees, and they suggested that their findings may shed light on the efficacy of various physical treatments in OA management [34]. The same authors suggested that LLLT was not the ideal treatment modality for OA. This contrasts with our findings that TNFα protein was reduced in both groups treated with LLLT, although only in the 100 mW group was this statistically significant. However, establishing an accurate comparison with the study performed by Guo and colleagues is difficult since the authors reported LLLT as follows: 'LLLT for 10 min: wavelength of 810 nm, the output power of the laser ranged from 0 mW to 1000 mW and could be adjusted by a controller. The radius of laser spot was approximately 5 mm with a distance of 1 cm between the surface of the knee and the lens in all the treatments' [34]. With this description is impossible to know the mean power output and consequently also the power density, energy density and energy delivered.

We note that the energy density (4 J·cm^2^) was the same for both groups (50 mW and 100 mW), but the irradiation time was twofold longer in the 50 mW group. We infer that this latter difference weighed importantly in the results. This assertion is supported by the results of the study by Castano and colleagues, who demonstrated that a longer lighting period is more effective than short times, regardless of the total influence or irradiance [35].

Our results provide evidence of LLLT-dependent reduction of IL-1β, IL-6 and TNFα, and the therapy's ability to inhibit proliferation of inflammatory cells makes it a suitable treatment for synovitis associated with the early stages of OA. The results also indicate that a better understanding of the role of LLLT in modulating these mediators can provide the basis for future therapeutic interventions.

## Conclusion

Despite both power outputs tested in this study showing positive results, LLLT with 50 mW was more efficient in modulating inflammatory mediators (IL-1β, IL-6) and inflammatory cells (macrophages and neutrophils), and led to histological signs of an attenuated inflammatory process.

## Abbreviations

OA: osteoarthritis; LLLT: low-level laser therapy; IL: interleukin; TNF: tumor necrosis factor; PBS: phosphate-buffered saline; PCR: polymerase chain reaction.

## Competing interests

ECPLJ receives research support from Multi Radiance Medical (Solon, OH - USA), a laser device manufacturer. Multi Radiance Medical had no role in the planning of this experiment, and the laser device used was not theirs. They had no influence on study design, data collection and analysis, decision to publish, or preparation of the manuscript. The remaining authors declare that they have no competing interests.

## Authors' contributions

ACAA, PdTCdC and RdPV contributed to the conception and design, acquisition of data, analysis and interpretation of data and drafting the manuscript. SAdS, ECPLJ, APL and RA were involved in data interpretation, statistical analysis and manuscript preparation. PdTCdC and JASJ conceived the study, participated in its design and coordination, and helped to draft the manuscript. All authors contributed to revising the manuscript critically for important intellectual content, and read and approved the manuscript for publication.
